# Dosimetric characteristics of 6MV flattening filter free and flattened beams among beam-matched linacs: a three-institutional study

**DOI:** 10.1186/s13014-023-02313-5

**Published:** 2023-07-28

**Authors:** Diana M. Ghemiș, Loredana G. Marcu, Vasile Virag, Adriana Virag

**Affiliations:** 1 Faculty of Physics, West University of Timisoara, Timisoara, Romania; 2 MedEuropa, Oradea, 410191 Romania; 3 Faculty of Informatics & Science, University of Oradea, Oradea, 410087 Romania; 4 UniSA Allied Health & Human Performance, University of South Australia, Adelaide, SA 5001 Australia

**Keywords:** Beam matched linacs, FFF beam, Dosimetry, Commissioning

## Abstract

**Background:**

Beam matching is a concept in radiotherapy applied to clinics where more than one linac is employed to harmonise beam characteristics across linacs for allowing patients interchange without replanning. In view of this, the current study analyzes and compares dosimetric characteristics of 6MV flattening filter free and flattened beams of three beam-matched linear accelerators (linacs) from three different clinics with the aim to evaluate the matching under tight criteria for gamma analysis.

**Methods:**

Three Elekta linacs from three different clinics were included. The linacs have the same collimator assembly, Elekta Agility. Beam data were collected during commissioning process using PTW dosimetry systems. Dose profiles and percentage depth doses (PDD) were analyzed using 1D gamma analysis (1 mm/1%) as well as the following parameters: depth of maximum dose, PDD10, flatness, unflattnes, symmetry, penumbra, output factors. Additionally, five stereotactic treatment plans were optimized in one clinic and calculated by all three planning systems (Monaco) for a dosimetric comparison.

**Results:**

Gamma analysis of dose profiles and PDDs showed clinically acceptable results of 96.3% passing rate for profiles and 100% passing rate for PDDs. All dosimetric parameters were in good agreement with the reference data. Furthermore, dosimetric comparisons between stereotactic treatment plans showed a maximum standard deviation of 0.48 Gy for the maximum dose to PTV, and a maximum standard deviation of 0.1 Gy for the dose to the organs at risk.

**Conclusions:**

All three linacs showed a strong agreement between parameters and passed the gamma analysis using 1% DD/1mm DTA criteria. This study confirmed the matching between linacs, offering the possibility to interchange patients with no replanning.

## Background

Beam matching is a common practice in clinics with more than one linear accelerator (linac) [1]. This concept brings important benefits for the medical team (medical physicists, physicians) and patients alike. Beam matched linacs are defined in the treatment planning system (TPS) using the same beam model (including parameters like leaf transmission, leaf leakage), and allow to calculate, deliver and perform pre-treatment verifications on any of the linacs included in the cohort with no additional labor for the medical team. For Elekta beam matched linacs, the TPS beam model is provided by the vendor and during the acceptance testing the linac beam is shaped to be in agreement with the TPS beam. A specific subset of beam profiles and percentage depth dose measurements are performed in order to verify the matching for broad square fields (typically 10 × 10cm^2^ and 30 × 30cm^2^). The agreement between the compared dosimetric data confirms that a single beam model could be used for the cohort of linacs involved [2–4]. Beam-matched linacs that belong to different clinics offer equivalent treatment parameters and allow for patient transfer between linacs/centers in case of machine break down. When dealing with a high patient throughput, the possibility of moving patients among linacs is highly desirable and recommended. Research into the degree of beam-matching between linacs has been in focus over the last years, particularly for stereotactic treatments. The accuracy of treatment delivery between beam-matched linacs was reported by several studies [2–5].

Some advantages of beam matching include the flexibility of interchanging patients between centers without replanning, and the establishment of analogous (equivalent) quality assurance procedures (monthly, semestrial and annual dosimetric measurement; pre-treatment QA) that correspond to an internal audit. An internal audit is a concept meant to check, in this case, the applicability of some procedures used for dosimetric measurements on beam matched linacs among different clinics which are involved in the study. In our country, as a legal requirement in the field of radiotherapy, remote dosimetry audits for high energy photon beam reference dosimetry are performed every 2 years, as per IAEA recommendations [6, 7]. Sustainable and systematic auditing programs are still needed at national level in many European countries with less than 50% rate of participation in dosimetry audits of Western Europe radiotherapy centers [6, 8, 9], result supported by European surveys [10]. Absolute dosimetry audits are, nevertheless, not sufficient for complex treatment verifications delivered via intensity modulated radiation therapy (IMRT) and volumetric modulated arc therapy (VMAT). To accommodate this, internal audits are designed to check dosimetric parameter similarities between linacs that might have an impact on treatment planning.

Elekta (Crawley, UK) linacs are able to deliver a variety of treatment techniques from conformal radiotherapy (3D-CRT) to volumetric modulated arc therapy (VMAT), including stereotactic radiotherapy using flattening filter free beams (FFF).

The Elekta’s commissioning process requires measurements for both small and large fields (3 × 3cm^2^, 5 × 5cm^2^, 10 × 10cm^2^, 30 × 30cm^2^ and 30 × 40cm^2^). While protocols for the analysis of reference field size 10 × 10cm^2^ and larger fields exist (IAEA TRS398 [11] and AAPM TG51 [12]), for small fields there is only one new protocol developed by IAEA and AAPM (TRS 483) [13] where dosimetric characteristics of small FFF fields are approached. In the literature there is only limited data, particularly for Elekta linacs, therefore, our study brings a contribution on FFF beams and small fields in terms of beam characteristics analysis.

Only linacs able to deliver both FFF and FF beams were included. The use of FFF beams is encouraged for stereotactic treatments by the beam properties (high dose rate, reduced treatment time, reduced head scatter, smaller leakage, lower out-of-field dose) as reported in the scientific literature [14]. There is a lack of information on FFF beams probably due to their relatively new implementation in radiotherapy, therefore this study presents a series of useful parameters that are also compared with reported data from the limited scientific literature.

The aim of the current study is to analyze and compare dosimetric characteristics of flattened and unflattened (FFF) photon beams of 6MV energy originating from three beam-matched linacs from three different centers. Beam data collected from linacs belonging to three different clinics was compared with the Elekta reference data, the study being designed as an internal audit.

Furthermore, pre-treatment specific QA for given IMRT/VMAT plans was performed as part of the commissioning process to ensure the beam matching between linacs and to check the possibility to deliver the same plan in any of the involved linacs with minimum deviations. Beam-matching is a concept also defined in the TPS, therefore, a dosimetric comparison of some complex treatment plans (stereotactic treatments) was performed.

## Materials and methods

### Linear accelerators

The current study includes 3 linacs installed in three different clinics across the country: one Elekta Versa HD (Linac 1) and two Elekta Infinity (Linacs 2 and 3), all equipped with the Agility multileaf collimator system and with the capacity to deliver both flattened and unflattened 6MV energy beams. The linacs are configured with the Agility multileaf collimator assembly (Elekta, UK), designed with 160 tungsten leaves of 0.5 cm width, the high speed of the leaf being the key characteristic of this assembly, with 6.5 cm/sec, possible only when leaves move in the same direction as the carriage. Real time positioning of the leaf is possible with Rubicon optic technology [15]. The radiation field size is defined by a pair of sculpted diaphragms, orthogonal to the MLC block. The MLCs replace the jaws in the orthogonal direction and there are no backup jaws or diaphragms. Old linacs design has two pairs of jaws, one on each axis. On new linacs designed for rapid and accurate treatments (IMRT, VMAT, SRS, SBRT), jaws from X axis were removed to increase MLC speed. All three linacs can deliver both flattened and unflattened beams. In this study we referred to 6MV flattened (FF) photon beam and 6MV flattening filter free (FFF) photon beam, with respective dose rates of 500MU/min and 1200MU/min. For Elekta accelerators, beam matched linacs have identical technical characteristics and multileaf collimator features.

### Commissioning

Photon beams are matched using beam profiles and the Percentage Depth Dose at 10 cm (PDD_10_) in SSD set-up at 90 cm. The beam matching process provided be Elekta is a fast process of acceptance and commissioning which allows clinical release within 2 weeks. The Elekta’s commissioning process requires measurements for the following field sizes: 3 × 3cm^2^, 5 × 5cm^2^, 10 × 10cm^2^, 30 × 30cm^2^ and 30 × 40cm^2^ (for wedged fields). The reference field size is 10 × 10cm^2^ measured at 10 cm depth. The accepted tolerances for 10 × 10cm^2^ field size for PDD10 must be within 1% and for 30 × 30cm^2^ within 2%. Measured data for PDDs and dose profiles underwent 1D gamma analysis using 1 mm distance to agreement (DTA) and 1% dose difference (DD) gamma passing criteria (stricter than required by the vendor) with the aim to check beam matching between machines, as an internal audit. The evaluated parameters include: flatness, symmetry, penumbra, depth of maximum, percentage depth dose at 10 cm, OFs based on data collected during commissioning of Elekta linacs.

To facilitate the dosimetric comparison and data analysis, data provided by Elekta was used as reference. Matching criteria required by the vendor is ± 1% from baseline for PDD and ± 2% from baseline for profiles. Beam adjustments required during calibration were performed by service engineers under TG 142 [16] protocol limits for stereotactic radiosurgery (SRS) and stereotactic body radiotherapy (SBRT).

### Measurements

Photon beams delivered by the three linacs underwent beam analysis for flatness, symmetry, penumbra, PDD_10_ and depth of maximum dose. Flattened beams were included in this study to highlight beam matching characteristics for situations where FFF beams cannot be used, especially when stereotactic treatments are optimized and delivered using standard photon beams.

Profiles, PDDs, and output factors (OF) were acquired with PTW Beam Scan (PTW Freiburg, Germany) at SSD 90 cm set-up. Mephysto mc^2^ software was used for data analysis according to the Elekta protocol predefined in the software. Profiles were measured at 5 cm, 10 and 20 cm depth for all field sizes.

To assess the consistency of dosimetric characteristics of the three linacs, three square fields were analyzed: 3 × 3cm^3^, 5 × 5cm^2^, 10 × 10cm^2^, the latter being the reference field based on TRS 398 [11] and TG 51 [12] recommendations. TRS 483 Code of Practice [13] defines new parameters of reference in small field dosimetry, such as that 3 × 3cm^2^ fields are defined as small fields and require special protocols for evaluation. Beam calibration was made with 100MU corresponding to 1 Gy at 10 cm depth and 90 cm SSD (SAD 100 cm) for 10 × 10cm^2^ field size.

Percentage depth dose values were acquired at 90 cm SSD for all field sizes: 3 × 3cm^2^, 5 × 5cm^2^ and 10 × 10cm^2^ using PTW 31021 Semiflex 3D ionization chamber positioned at the effective point of measurement with 1 mm detector step. PTW 31021 Semiflex 3D ion chamber was employed also as reference detector.

For profile measurements a 2 mm measurement step was used for the central region and the out of field region and a 1 mm step for the penumbra region. Profiles were measured for X (cross-plane) and Y (in-plane) axis at three depths: 5 cm, 10 cm and 20 cm, at 90 cm SSD using PTW 31021 Semiflex 3D ionization chamber as reference detector, normally placed in air at radiation field edges with the aim to suppress fluctuations in signal acquisition and saturation effects for small fields. The same PTW 31021 Semiflex 3D Ion Chamber was employed for 5 × 5cm^2^ and 10 × 10cm^2^ field sizes while for 3 × 3cm^2^ the PTW 60019 microDiamond and PTW 60023 microSilicon were used.

The output factors (OF) were measured at 90 cm SSD and 10 cm depth using PTW 31021 Semiflex 3D ionization chamber. For small fields (3 × 3cm^2^) a second measurement was conducted using PTW 31022 PinPoint 3D detector to eliminate perturbation errors due to detector volume.

All measurements reported in this study were performed in a PTW BeamScan (Freiburg) water phantom and for detector positioning inside the phantom a PTW TruFix (Freiburg) dedicated system was used. All detectors used for commissioning are recommended by International Codes of Practice: TRS 398 (IAEA) [11], TG 51 (AAPM) [12], TRS 483 (IAEA and AAPM) [13].

### Beam data analysis

#### PDDs

Depth of maximum and PDD at 10 cm depth were the main characteristics analyzed for this study due to their impact on the treatment planning process. For commissioning, R80 that corresponds to the 80% of the PDD is also used to define the therapeutic range of the beam and the quality index of the beam (Q_i_). For depth of maximum parameter, a maximum deviation of 1 mm from baseline is admitted and 1% deviation for PDD_10_.

#### Dose profiles

For dose profiles evaluation, flatness and symmetry are the main characteristics of a beam determined from the central region of the profile. For FFF beams the literature highlights as main characteristic the central peak (unflatness), determined using the renormalization factor which is defined as the relative dose ratio in the central axis of the beam (D_CAX_) when the two field edges corresponding to the FFF and FF beams overlap [17, 18]. Mephysto mc^2^ software for relative dosimetry analysis defines unflatness as the ratio between D_CAX_ and D_Xoff−axis_, (according to the Elekta protocol), where D_CAX_ is the dose at central axis and D_Xoff−axis_ is the dose at offset calculated with formula (1) for field sizes < 10 × 10cm^2^ and with formula (2) for field sizes ≥ 10 × 10cm^2^:

1 $$\frac{Field size}{2}x60\%$$

2 $$\frac{Field size}{2}x80\%$$

Symmetry is defined as the evenness between two points from the profile situated at the same distance from the central axis. Profile symmetry analyzed by Mephysto software is calculated based on IEC 60976 international standards using formula (3):

3 $$Symmetry= \left(\frac{{D}_{\left(x\right)}}{{D}_{(-x)}}\right)x100$$

where D(x) is the point dose at a given distance from the central axis.

For unflatness, flatness and symmetry, 1% is the allowed tolerance for the analyzed field sizes as a study criterion. The vendor allows a 2% deviation from baseline for dose profile analysis.

Penumbra variations are related to the radiation field size and the presence of jaws in the X axis over the MLC block. On new linacs designed for rapid and accurate treatments (IMRT, VMAT, SRS, SBRT), jaws from X axis were removed to increase MLC speed. Beside the benefits brought by jaw removal, cross-plane penumbra shows increased values.

For conventional flattened beams, penumbra is defined as the distance between the 80% and 20% isodose lines normalized to the dose at central axis [19, 20]. In case of FFF beams, due to penumbra changes as a consequence of flattening filter removal, the standard definition of penumbra is not valid anymore. Ponisch et al. [17] proposed a new method for penumbra definition for FFF beams which involves the use of inflection points at the field edge for FFF beam normalization at the same dose level of a flattened beam. If FFF beam profiles are normalized to the same dose level as the flattened beam - a method proposed by Fogliata et al. [21], penumbra evaluation based on the 80 − 20% dose values of the profile in the field edges is accepted. As the involved linacs are also used for stereotactic treatments, the deviation of penumbra allowed from baseline is 1 mm.

#### Output factors

The output factors (OF) are defined as the ratio between the absorbed dose in water for any field size and reference field size (10 × 10cm^2^) at reference depth. The tolerance is 1% for 5 × 5cm^2^ and 10 × 10cm^2^, while for 3 × 3cm^2^ the tolerance is 2%.

#### Gamma analysis

Gamma analysis employed in the dosimetric evaluation incorporates two criteria: distance-to-agreement (DTA) and dose-difference (DD), in one analysis, covering both low and high dose gradient regions [22, 23].

For this study, measured PDDs and profiles underwent a 1D gamma analysis where gamma criteria were reduced to 1%/1mm with the aim to achieve higher standards and to minimize the error that can appear for small fields analysis due to varying perturbation effects.

### IMRT/VMAT-specific PSQA

In a standard workflow of a commissioning process, the next step after beam data collection is beam modelling. An accurate beam modelling is mandatory for beam matched linacs and can take several weeks. By choosing Accelerated Go Live process for acceptance and commissioning, Elekta provides a set of beam models, the selected data sets being in accordance with the TG-119 protocol [24, 25]. Model validation process is part of commissioning process and includes a set of IMRT and VMAT treatments plans given by Elekta, that undergo calculation in the local TPS, without optimization. Plans are delivered and analyzed using gamma analysis with 3 mm distance to agreement and 3% dose difference criteria as per TG218, as the commissioning of the linacs was performed before the release of the TG219 protocol in 2021 [26]. Test plans covered a range of anatomical sites (brain – 2 plans, head and neck – 3 plans, pelvis – 2 plans) and they are delivered using all available energies.

Quality assurance procedures employed the PTW Octavius 4D rotation unit which can perform 3D dose verification including non-coplanar beams, off-axis target volumes and very large fields. For IMRT/VMAT plan validation, PTW Octavius Standard Top together with PTW Octavius 1500 Detector were used [27].

### Treatment planning evaluation

The commissioning process involves a post modelling feature where different IMRT and VMAT plans provided by vendor are delivered and compared. However, stereotactic plans can be considered as a deficiency of fast Elekta commissioning process, due to the fact that beam matching among linacs that are able to deliver stereotactic treatments need special consideration. The smallest radiation field measured is 3 × 3cm^2^. Nevertheless, this field size is not small enough to thoroughly evaluate a linac stereotactically.

To undertake a planning comparison between the three linacs, five stereotactic treatment plans for brain metastases were calculated without optimization by the three planning systems. In the TPS, the machine characteristics is predefined by the vendor, but there are some adjustments made for each machine concerning leaf leakage and leaf transmission that are consistent with the measurements employed for IMRT/VMAT validation during the commissioning process.

Additionally, this study also includes a clinical evaluation of five stereotactic treatments with treatment delivery involving small fields. All selected treatment plans delivered hypofractionated stereotactic radiotherapy (HSRT) with a total dose of 30 Gy delivered in 5 fractions (6 Gy/fr). The calculation algorithm was Photon Monte Carlo (pMC) using the same calculation parameters (0.5% uncertainty) in Monaco 5.51 TPS version. In terms of target, three volumes were contoured for each case: gross target volume (GTV), clinical target volume (CTV) and planning target volume (PTV). The isocenter was placed in the geometric center of the GTV established by the TPS. For all cases, coplanar and non-coplanar beams were used. All patients were treated with FFF beams via volumetric modulated arc therapy (VMAT) using partial arcs. The arc length, couch angle and collimator angle were established by the medical physicist according to patient anatomy and tumor position also accounting for the organs at risk (OARs). The number of treatments fields (arcs) was directly correlated with the complexity of the plan and increases for the cases requiring high dose sparing.

The five stereotactic treatment plans optimized in Monaco TPS in one of the three clinics were transferred to the other TPSs and calculated without optimization using the characteristics of the linacs involved. The plans were dosimetrically analyzed with the aim to achieve results within a 3% dosimetric difference.

## Results

### Percentage depth dose

The value of d_max_ for FFF beams was greater than that of corresponding flattened beams as expected, shifted from 15 mm for 6MV flattened beam to 17 mm for 6MV FFF (Table 1). Beam intensity for FFF beams was increased owing to filter removal from the beam trajectory. For Elekta linacs, each FFF energy had its own settings, different from any flattened beams allowing to restore the penetrative quality of the FFF beams to the nominal value. For 6MV flattened beam, d_max_ variation had a maximum of 0.69 mm from baseline (reference data). For 6MV FFF beam the maximum deviation was 0.82 mm from baseline for a 10 × 10cm^2^ field size. PDD at 10 cm depth (PDD_10_) in water increases proportionally with the field size for both beam types.

By superimposing PDDs of the same field size for both flattened and unflattened beams, more than one point overlapped in the region of d_max_, called plateau. For 6MV FFF beams, the region of plateau was seen around 1.7 cm depth extending with maximum 1 mm.

**Table 1:** Depth of maximum dose and PDD at 10 cm depth for 6MV flattened and unflattened beams* (reproduced with permission from MedEuropa)*

Beam type	6MV FFF			6MV		
Field size (cm^2^)	3 × 3	5 × 5	10 × 10	3 × 3	5 × 5	10 × 10
d_max_ (mm)						
Reference	16.97	16.99	16.98	15.01	15.01	14.99
Linac 1	16.6	17.3	17.8	14.9	15.7	15.0
Linac 2	17.0	17.2	16.4	15.7	15.7	15.1
Linac 3	16.9	17.2	17.0	15.0	15.5	15.1
PDD_10_ (%)						
Reference	60.96	62.95	65.9	60.49	62.54	65.95
Linac 1	60.58	62.91	66.03	60.21	62.86	65.86
Linac 2	60.76	62.8	65.95	60.61	62.7	65.95
Linac 3	60.93	62.88	65.96	60.57	62.46	65.95

### Dose profiles

Unflatness values for field sizes smaller than reference (3 × 3cm^2^ and 5 × 5cm^2^) were similar for both cross-plane and in-plane axis, while for 10 × 10cm^2^ unflatness was significantly increased, as expected due to field size dependence (Table 2). Maximum deviation from baseline was within the limit of 1% imposed by this study with one exception, at the 3 × 3cm^2^ field size, depth 200 mm where for Linac 1, the deviation was  +1.11% but still within the limit requested by the vendor (± 2%).

**Table 2:** Unflatness and symmetry for 6MV FFF beam measured at three different depth 5 cm, 10 cm and 20 cm. *(reproduced with permission from MedEuropa)*

Parameter	Field size (cm^2^)	Depth (mm)	Ref.	Measured data			Deviation from reference (%)		
				Linac 1	Linac 2	Linac 3	Linac 1	Linac 2	Linac 3
Unflatness	3x3	50	1.035	1.043	1.038	1.038	0.77	0.24	0.29
	3x3	100	1.034	1.043	1.038	1.039	0.87	0.39	0.44
	3x3	200	1.033	1.044	1.039	1.040	1.11	0.58	0.73
	5x5	50	1.036	1.037	1.036	1.037	0.05	0.00	0.05
	5x5	100	1.039	1.041	1.038	1.039	0.19	0.10	0.0
	5x5	200	1.040	1.041	1.040	1.040	0.05	0.05	0.00
	10x10	50	1.134	1.134	1.132	1.135	0.00	-0.13	0.13
	10x10	100	1.148	1.148	1.147	1.150	0.00	-0.09	0.22
	10x10	200	1.159	1.157	1.158	1.159	-0.17	0.13	0.04
Symmetry	3x3	50	100.00	100.44	100.15	100.51	0.44	0.15	0.51
	3x3	100	100.00	100.33	100.20	100.43	0.33	0.20	0.43
	3x3	200	100.00	100.52	100.43	100.51	0.52	0.43	0.51
	5x5	50	100.00	100.21	100.23	100.28	0.21	0.23	0.28
	5x5	100	100.00	100.32	100.20	100.29	0.32	0.20	0.29
	5x5	200	100.00	100.29	100.25	100.22	0.29	0.25	0.22
	10x10	50	100.00	100.43	100.41	100.32	0.43	0.41	0.32
	10x10	100	100.00	100.67	100.38	100.37	0.67	0.38	0.37
	10x10	200	100.00	100.55	100.38	100.28	0.55	0.38	0.28

Flattened beams presented an average deviation of flatness from reference data of ± 0.42% with higher values for small fields (average ± 1.09%) due to perturbation factors involved in small field measurement. Cross plane dose profiles showed higher deviations and need some beam profile adjustments available in the measurement software, such as smoothing functions and beam normalization to CAX (central axis deviation of the profile). Table 3 tabulates only the results for 10 × 10cm^2^ field size.

**Table 3:** Flatness and symmetry for 6MV beam field size 10 × 10cm^2^, measured at three different depth 5 cm, 10 and 20 cm. *(reproduced with permission from MedEuropa)*

Parameters	Depth (mm)	Ref.	Measured data			Deviation from reference (%)		
			Linac 1	Linac 2	Linac 3	Linac 1	Linac 2	Linac 3
Flatness	50	102.36	102.38	102.33	102.47	0.02	-0.03	0.11
	100	104.16	104.28	104.25	104.22	0.12	0.09	0.06
	200	105.91	106.10	105.93	106.06	0.17	0.02	0.14
Symmetry	50	100.00	100.36	100.26	100.53	0.36	0.26	0.53
	100	100.00	100.42	100.31	100.50	0.42	0.31	0.50
	200	100.01	100.46	100.43	100.48	0.46	0.42	0.47

Reference symmetry values showed a perfect symmetric profile from central axis. All measured profiles for FFF beams were consistent with deviations allowed by protocols, showing increased values for symmetry in cross-plane with maximum deviation of 0.9%. For reference field size, cross-plane (X axis) and in-plane (Y axis), symmetry values for FFF beams were comparable to their flattened counterparts (Table 3). For an easier comparison of key parameters such as flatness, symmetry and penumbra, measured values at 10 cm depth for 10 × 10cm^2^ reference field are tabulated in Table 4.

**Table 4:** Flatness, symmetry and average penumbra for 10 × 10cm^2^ reference field measured at 90 cm SSD and 10 cm depth *(reproduced with permission from MedEuropa)*

LINAC	Beam type	Unflatness and Flatness (%)		Symmetry (%)		Average penumbra (mm)	
		in-plane	cross-plane	in-plane	cross-plane	in-plane	cross-plane
Linac 1	6MV	103.9	104.66	100.39	100.44	6.43	7.9
	6MV FFF	1.142	1.153	100.43	100.9	6.33	7.57
Linac 2	6MV	103.9	104.6	100.38	100.24	6.44	7.88
	6MV FFF	1.142	1.151	100.27	100.49	6.34	7.59
Linac 3	6MV	103.82	104.62	100.51	100.49	6.45	7.77
	6MV FFF	1.147	1.153	100.58	100.16	6.35	7.64

As expected, average penumbra (Table 5) in cross-plane was slightly increased towards in-plane for both beam type, with + 1.3 mm in the case of FFF beams and + 1.45 mm for flattened beams. All linacs analyzed in this study shows small differences in penumbra for FFF beams, with an average of -0.15 mm smaller than for flattened beams, as indicated in Table 5.

**Table 5:** Average penumbra (mm) for 6MV flattened and unflattened beams *(reproduced with permission from MedEuropa)*

Beam type	Field size (cm^2^)	Right Penumbra (mm)				Left Penumbra (mm)			
		Reference	Linac 1	Linac 2	Linac 3	Reference	Linac 1	Linac 2	Linac 3
6MV FFF	3 × 3	4.27	4.77	4.46	4.36	4.27	4.70	4.43	4.39
	5 × 5	6.39	6.40	6.11	6.13	6.39	6.38	6.12	6.13
	10 × 10	7.33	7.25	7.23	7.27	7.33	7.16	7.23	7.26
6MV	3 × 3	4.39	4.75	4.57	4.46	4.39	4.72	4.52	4.47
	5 × 5	6.51	6.51	6.34	6.34	6.51	6.49	6.33	6.31
	10 × 10	7.53	7.48	7.48	7.42	7.53	7.46	7.42	7.43

### Output factors

In this study, the output factor ranged from 0.874 to 1.093 for 6MV FFF beams and from 0.845 to 1.149 for 6MV flattened beams (Table 6).

**Table 6:** Output factors for 6MV flattened and unflattened beams *(reproduced with permission from MedEuropa)*

Beam type	Field size (cm^2^)	Output factor			
		Reference	Linac 1	Linac 2	Linac 3
6MV FFF	3 × 3	0.874	0.874	0.874	0.874
	5 × 5	0.927	0.927	0.926	0.926
	10 × 10	1	1	1	1
6MV	3 × 3	0.846	0.845	0.845	0.845
	5 × 5	0.906	0.906	0.904	0.905
	10 × 10	1	1	1	1

### Gamma analysis

For dose profile assessment 54 gamma analyses was undertaken for FFF beams and the same number for flattened beams (a total of 108 dose profiles) with clinically acceptable results of 96.3% passing rate for both cases. The assessment of FFF beams showed two dose profiles that need different gamma criteria to pass the analysis: for Linac 3, 10 × 10cm^2^ field size at 5 cm depth, Y axis profile, 1%/1mm criteria shows one point dose from the edge of the field to be situated at the limits and for the same linac, the 10 × 10cm^2^ field size at 20 cm depth on Y axis, only met the limit vendor requirement of 2%/2mm criteria for 2 dose points. Flattened beams presented deviation from the standard criteria on two profiles for 3 × 3cm^2^ field size along Y at 5 and 10 cm depth; one point in each case needed a 2% dose-difference criteria to pass the analysis. All measured PDDs passed the gamma analysis using a 1%/1mm criteria for all measured points.

### IMRT/VMAT plan validation

Gamma passing rates using 3%/3mm (according to TG119) criteria showed good agreement between machines. All measured plans met the minimum passing rate required in our clinics of 95%. Linac 1 showed the best results in terms of passing rates with minimum of 98.4% (Table 7). The average gamma passing rate for IMRT plans was 98.9% for Linac 1, 97.2% for Linac 2 and 98.3% for Linac 3; and for VMAT plans 99.10% for Linac 1, 96.87% for Linac 2 and 97.46% for Linac 3.

**Table 7:** Gamma passing rate for 6MV FFF photon beam IMRT / VMAT plan

Treatment plan	Linac 1	Linac 2	Linac 3
C119 IMRT	99.2%	97.2%	99.1%
HEAD AND NECK 119 IMRT	98.6%	97.2%	97.5%
ANAL VMAT	99.6%	97.2%	96.9%
C VMAT	98.4%	95.1%	96.1%
HEAD AND NECK 119 VMAT	98.8%	95.6%	97.1%
HEAD AND NECK 244 VMAT	99.4%	98.4%	97.3%
PROSTATE VMAT	99.7%	97.4%	98.2%
**Average gamma passing rate**	99.10%	96.87%	97.46%

### Treatment planning evaluation

Stereotactic treatment plan evaluation for five brain metastases showed small differences between linacs, with a maximum standard deviation of 0.48 Gy for the maximum dose to PTV, and a maximum standard deviation of 0.1 Gy for the dose to the organs at risk and PTV coverage.

## Discussions

This study reported the dosimetric compatibility and consistency of three Elekta beam matched linear accelerators (Versa HD and Infinity) from three radiotherapy centers. Beam matched linacs bring benefits to both medical team and patients alike [29], enabling treatment on any of the devices without requiring re-planning. This aspect is of real assistance when a linac is unavailable or when large number of patients are awaiting radiotherapy.

While this topic presents interest in radiotherapy, it is under-researched especially in Eastern European countries. The concept of dosimetric compatibility for FFF beams is even less approached. A PubMed search on “beam matched linac FFF” displayed only 19 articles published between 2011 and 2022, with 8 studies referring to Varian TrueBeam or Halcyon accelerators and 5 to Elekta linacs, whereas the other studies did not include physical parameters.

To be noted that all measurements within this study were carried out on Elekta machines. Despite differences in design and characteristics of the treatment head between Elekta and Varian linacs, the main parameters were also correlated with results from Varian machines. This was done in order not to limit the results of our study to Elekta linacs but to provide a methodology that is applicable to all linacs.

For dosimetrically compatible linear accelerators Elekta requires a tolerance of ± 1% for PDDs and ± 2% for dose profiles. A report of the Elekta’s Accelerated Go Live commissioning process shows a 95% compatibility for gamma analysis of PDD with a criterion of 1% dose and 1 mm distance, and 2% dose and 2 mm distance for dose profiles [30].

Unflattened beams often represent a challenge in radiotherapy, particularly for medical physicists responsible for dosimetric quality assurance. A new Code of Practice, TRS 483, developed by IAEA and AAPM, give information about small fields dosimetry delivered by FFF beams to assist physicists with this task [29].

The maximum depth for 6MV standard photon beam is 1.5 cm, in agreement with the values reported in the literature [31] and also with Monte Carlo simulations of a linear accelerator that operates with both flattened beams and FFF beams [32]. The d_max_ value of the 6MV FFF photon beam is 2 mm deeper than for the 6MV flattened photon beam, thus being consistent with the d_max_ value of the reference FFF photon beam provided by the manufacturer Elekta and within the accepted deviation limits of 1 mm compared to data reported in the literature on Elekta linacs [19, 33].

The dose deposited by the standard beam at 10 cm depth (PDD10 or D100) is consistent with the data published in the literature for both standard and unflattened beams, for all field sizes. PDD_10_ for FFF beams has a maximum difference of 1.7% and 1.3% for flattened beams from the reported data including both Elekta and Varian linacs (Table 8) [19, 25, 34–36].

**Table 8:** Penumbra variations for flattened and unflattened beams between our study and the scientific literature

Study (ref)	Beam type	Field size (cm^2^)	Depth (mm)	Reported value (mm)	Differences from our measurements (mm)		
					Linac 1	Linac 2	Linac 3
**Elekta Versa HD** (Narayanasamy et al. 2016)	6MV FFF cross-plane	10 × 10	100	7.2	+ 0.37	+ 0.39	+ 0.44
	6MV FFF in-plane			5.1	+ 1.23	+ 1.24	+ 1.25
	6MV FF cross-plane			7.6	+ 0.30	+ 0.28	+ 0.17
	6MV FF in-plane			5.5	+ 0.93	+ 0.94	0.95
**Elekta Versa HD** (Meshram et al., 2016)	6MV FFF	10 × 10	100	7.9	-0.95	-0.94	-0.91
	6MV FF			8.4	-1.24	-1.25	-1.29
**Varian Clinac 21EX and MCNPX Monte Carlo Simulations** (Ponisch et al., 2006)	6MV FFF measured cross-plane	10 × 10	100	6.4	+ 1.17	+ 1.19	+ 1.24
	6MV FFF measured in-plane			5.2	+ 1.13	+ 1.14	+ 1.15
	6MV FFF simulations cross-plane			5.9	+ 1.67	+ 1.69	++1.74
	6MV FFF simulations in-plane			5	+ 1.33	+ 1.34	+ 1.35
	6MV FF measured cross-plane			7.2	+ 0.70	+ 0.68	+ 0.57
	6MV FF measured in-plane			6	+ 0.43	+ 0.44	+ 0.45
	6MV FF simulation cross-plane			6.4	+ 1.50	+ 1.48	+ 1.37
	6MV FF simulation in-plane			5.9	+ 0.53	+ 0.54	+ 0.55
**Varian TrueBeam** (Beyer, 2013)	6MV FF	10 × 10	100	5.7	+ 1.46	+ 1.46	+ 1.41
				5.6	+ 1.56	+ 1.56	+ 1.51
				5.5	+ 1.66	+ 1.66	+ 1.61
**Varian TrueBeam** (Tanaka et al., 2020)	6MV FFF	3 × 3	100	5.76	-1.13	-1.41	-1.48
		10 × 10		6.87	+ 0.08	+ 0.09	+ 0.12

Acronyms: FF = flattened beam; FFF = flattening filter free beam.

X axis penumbra values for both flattened and FFF beams, for all field sizes, regardless of depth, are higher than the in-plane axis penumbra values, owing to the removal of the X jaws to allow for fast and accurate treatment delivery. A comparative study regarding penumbra variation for both flattened and unflattened beams is presented in Table 8 which includes 2 Elekta Versa HD and 3 Varian Linacs. The average values ​​of the penumbra along X axis for both beam types are ​​at least 1 mm larger than the values along the in-plane axis, in agreement with reported data [17, 19]. For the reference field, the penumbra value for 6MV FFF photons along X is 1.27 ± 0.06 mm larger than the Y penumbra, while for the 6MV FF photons the penumbra in the X axis is 1.5 ± 0.11 mm larger than the penumbra value along Y. The method proposed by Fogliata et al. [21] for beam normalization together with penumbra evaluation based on the 80 − 20% dose values of the profile in the field edges shows no significant differences (lower than 1 mm) from the values reported by Ponisch et al. [17]

For 10 × 10cm^2^ field size, in the case of FF and FFF beams, average values of penumbra for all three linacs show less than 1 mm difference from the data reported by Meshram et al. [33] and Beyer et al. [37] (see also Table 4).

New parameters specific to FFF beams were proposed by Fogliata et al. [21], including unflatness. The data analyzed for all field dimensions at the three depths show a maximum deviation of -0.29% for the 3 × 3cm^2^ field in the X axis. Our measured data is in agreement with the data published by Fogliata et al. [21] with a small difference for the reference field of 0.21‰.

Tanaka et al. [38]. compared the impact of detector selection for FFF beam measurements for TrueBeam linacs. The unflateness variation is insignificant regarding selected detector. All measured values are in good agreement with the values measured for Elekta linacs involved in this study [38].

For standard beams, the flatness for reference field of 10 × 10cm^2^ measured at 10 cm depth is in accordance with data reported by Fogliata et al. [39] who shows that Elekta FF and FFF photon beams have similar characteristics.

The values obtained for FFF photon beam symmetry for a 10 × 10cm^2^ field in our study are ​​comparable to those reported in the literature, i.e., below 101%, both in the X and Y axis of the dose profile, while symmetry values for the standard beam for the same field size ​​are below the average value of 100.5% reported by Narayanasamy et al. [19].

The total scattering factor can be expressed as a function of the output factor, as the ratio between the absorbed dose at the reference depth for a given field size and the dose at the same depth for the 10 × 10cm^2^ reference field. The output factor values for the standard and FFF photon beam are in agreement with those reported by other studies [34, 40] for all field sizes.

Quality assurance procedures for beam characteristics and treatment planning during linac commissioning are critical aspects for beam-matched linacs. All IMRT and VMAT plans successfully passed the gamma criteria of 3% dose difference and 3 mm distance to agreement with an overall average of 97.81%. Similar results are reported by Snyder et al. [28] for a Versa HD linear accelerator using two identical plans. A high passing rate showed a good agreement between beam models and linac performance. In situations when the difference between linacs is higher than 3%, pre-treatment verification is recommended to be performed before patient interchange.

To be noted that stereotactic treatments need special consideration in terms of beam matching when interchanging patients. This is because high doses are delivered over a short period of time and in most cases, tumors are located around vital organs at risk. Therefore, beside beam data analysis, a complementary clinical data analysis was performed which included 5 stereotactic treatment plans. The impact of the presented difference can be considered negligible when switching patients between linacs and offers again the certainty that patients are treated with clinically insignificant deviations from the original plan.

## Conclusions

In this study we investigated the consistency of dosimetric characteristics among flattening filter free beam-matched linacs across three clinics focusing on flattening filter free beams due to lack of literature in this regard. Parameters such as flatness, symmetry, penumbra, PDD_10_ from profiles and percentage depth dose measurements were analyzed using Mephysto mc^2^ software according to international recommendations from IAEA, AAPM and vendor (Elekta). All data were collected during Elekta commissioning process.

Overall, all three linacs show a strong agreement between parameters. The analyzed dosimetric parameters are also consistent with the data reported by literature with clinical acceptable results in terms of IMRT/VMAT treatment plan validation (higher than 95%). Beam matching was also checked for five stereotactic treatment plans of brain metastases optimized in one planning system and then calculated for each linac involved in this study, showing a good agreement in DVHs parameters.

This interinstitutional study highlights the role of beam-matching between linacs and offers the possibility to interchange patients between linacs with no additional labor, such as replanning.

## Data Availability

The datasets used and/or analyzed during the current study are available from the corresponding author on reasonable request.

## List of abbreviations

3D-CRT: 3D conformal radiotherapy
AAPM: American Association of Physicists in Medicine
AGL: Accelerated Go Live
D100: Depth Dose at 10 cm
DD: Dose difference
DTA: Distance to agreement
FF: Flattened
FFF: Flattening filter free
IAEA: International Atomic Energy Agency
IMRT: Intensity-modulated radiation therapy
Linac: Linear accelerator
MLC: Multi-leaf collimator
MU: Monitor Unit
OAR: Organs at risk
OF: Output factor
PDD: Percentage depth dose
QA: Quality assurance
SAD: Source-to-axis distance
SBRT: Stereotactic body radiotherapy
SRS: Stereotactic radiosurgery
SSD: Source to surface distance
TPS: Treatment planning system
VMAT: Volumetric modulated arc therapy

## References

[1] Sjöström D, Bjelkengren U, Ottosson W, Behrens CF (2009). A beam-matching concept for medical linear accelerators. Acta Oncol.

[2] Rijken J, Schachenmayr H, Crowe S, Kairn T, Trapp J. Distributive quality assurance and delivery of stereotactic ablative radiotherapy treatments amongst beam matched linear accelerators: a feasibility study. J Appl Clin Med Phys. 2019 Apr;20(4):99–105. 10.1002/acm2.12567 10.1002/acm2.12567 PMC6448346 30883010

[3] Muñoz L, Kron T, Petasecca M, Bucci J, Jackson M, Metcalfe P, Rosenfeld AB, Biasi G. Consistency of small-field dosimetry, on and off axis, in beam-matched linacs used for stereotactic radiosurgery. J Appl Clin Med Phys 2021 Feb; 22(2):185–93. 10.1002/acm2.13160 10.1002/acm2.13160 PMC7882112 33440049

[4] Rojas-López JA, Venencia D. Importance of Beam-Matching between TrueBeam STx and Novalis Tx in Pre-Treatment Quality Assurance using Portal Dosimetry. J Med Phys. 2021 Jul-Sep;46(3):211–20. 10.4103/jmp.JMP_12_21 10.4103/jmp.JMP_12_21 PMC8491317 34703106

[5] Krishnappan C, Radha CA, Balaji K, Mani PK, Subramani V, Thanigaimalai V, Gunasekaran MK, Ramasubramanian V. Evaluation of beam matching accuracy among six linacs from the same vendor. Radiol Phys Technol. 2018 Dec;11(4):423–33. 10.1007/s12194-018-0480-3 10.1007/s12194-018-0480-3 30269310

[6] Izewska J, Lechner W, Wesolowska P. Global availability of dosimetry audits in radiotherapy: the IAEA dosimetry audit networks database. Phys Imaging Radiat Oncol. 2018 Jan;10:5:1–4. 10.1016/j.phro.2017.12.002 10.1016/j.phro.2017.12.002 PMC7807735 33458360

[7] National Commission For The Control Of Nuclear Activities., Romania, Radiological safety rules in the practice of radiotherapy; approved by CNCAN Order no. 94 /14.04.2004; [accessed May 2023], Available at: http://www.cncan.ro/assets/NSR/nsr-12-ordin-cncan-94-2004.pdf

[8] Dixon P, O’Sullivan B. Radiotherapy quality assurance: time for everyone to take it seriously. Eur J Cancer. 2003 Mar;39(4):423–9. 10.1016/s0959-8049(02)00744-x 10.1016/s0959-8049(02)00744-x 12751371

[9] van der Merwe D, Christaki K. IAEA support to radiotherapy dosimetry. Acta Oncol. 2020 May;59(5):493–4. 10.1080/0284186X.2020.1726457 10.1080/0284186X.2020.1726457 32056484

[10] Casares-Magaz O, Marcu LG, Prezado Y, Koutsouveli E, Brambilla M. EFOMP survey results on national radiotherapy dosimetry audits. Phys Med. 2021 Apr;84:10–4. 10.1016/j.ejmp.2021.03.020 10.1016/j.ejmp.2021.03.020 33799057

[11] Andreo P, Burns DT, Hohlfeld K, Huq MS, Kanai T, Laitano F, Smyth V, Vynckier S. Absorbed Dose Determination in External Beam Radiotherapy: An International Code Of Practice For Dosimetry Based On Standards Of Absorbed Dose To Water, IAEA, Vienna, 2000, Chap. 6. Code of practice for high energy photon beams.

[12] Almond PR, Biggs PJ, Coursey BM, Hanson WF, Huq MS, Nath R, Rogers D (1999). W.O. AAPM’s TG-51 protocol for clinical reference dosimetry of high energy photon and electron beams. Med Phys.

[13] Palmans H, Andreo P, Huq MS, Seuntjens J, Christaki KE, Meghzifene A (2018). Dosimetry of Small Static Fields used in External Photon Beam Radiotherapy: Summary of TRS 483, the IAEA-AAPM international code of practice for reference and relative dose determination. Med Phys.

[14] Ghemis DM, Marcu LG (2021). Progress and prospects of flattening filter free beam technology in radiosurgery and stereotactic body radiotherapy. Crit Rev Oncol Hematol.

[15] Cosgrove VP, Thomas MDR, Weston SJ, Thompson MG, Reynaert N, Evans CJ, Brown KJ, De Wagter C, Thwaites DI, Warrington AP (2009). Physical characterization of a New Concept Design of an Elekta Radiation Head with Integrated 160-leaf multi-leaf Collimator. Int J Radiat Oncol Biol Phys.

[16] Klein EE, Hanley J, Bayouth J, Yin FF, Simon W, Dresser S, Serago C, Aguirre F, Ma L, Arjomandy B, Liu C, Sandin C, Holmes T. Task Group 142, American Association of Physicists in Medicine. Task Group 142 report: quality assurance of medical accelerators. Med Phys. 2009 Sep;36(9):4197–212. 10.1118/1.3190392 10.1118/1.3190392 19810494

[17] Ponisch F, Titt U, Vassiliev ON, Kry SF, Mohan R (2006). Properties of unflattened photon beams shaped by a multileaf collimator. Med Phys.

[18] Yan Y, Yadav P, Bassetti M, Du K, Saenz D, Harari P, Paliwal BR (2016). Dosimetric differences in flattened and flattening filter-free beam treatment plans. J Med Phys.

[19] Narayanasamy G, Saenz D, Cruz W, Ha C, Papanikolaou N, Stathakis S (2016). Commissioning an Elekta Versa HD linear accelerator. J Appl Clin Med Phys.

[20] International Electrotechnical Commission. Medical electrical equipment. Part 2: Particular requirements for the safety of gamma beam therapy equipment. *IEC60601-2-11*, Geneva, 1987.

[21] Fogliata A, Garcia R, Knoos T, Nicolini G, Clivio A, Vanetti E, Khamphan C, Cozzi L. Definition of parameters for quality assurance of flattening filter free (FFF) photon beams in radiation therapy. Med Phys. 2012 Oct;39(10):6455–64. 10.1118/1.4754799 10.1118/1.4754799 23039680

[22] Low DA, Harms WB, Mutic S, Purdy JA. A technique for the quantitative evaluation of dose distributions. *Med Phys*. 1998 May;25(5):656 – 61. 10.1118/1.598248. PMID: 9608475. 10.1118/1.598248 9608475

[23] Harms WB, Sr, Low DA, Wong JW, Purdy JA. A software tool for the quantitative evaluation of 3D dose calculation algorithms. *Med Phys*. 1998 Oct;25(10):1830-6. 10.1118/1.598363. PMID: 9800688. 10.1118/1.598363 9800688

[24] Wen N, Zhao B, Kim J (2014). IMRT and RapidArc commissioning of a TrueBeam linear accelerator using TG-119 protocol cases. J Appl Clin Med Phys.

[25] Ezzell GA, Burmeister JW, Dogan N (2009). IMRT commissioning: multiple institution planning and dosimetry comparisons, a report from AAPM Task Group 119. Med Phys.

[26] Miften M, Olch A, Mihailidis D, Moran J, Pawlicki T, Molineu A, Li H, Wijesooriya K, Shi J, Xia P, Papanikolaou N, Low DA. Tolerance limits and methodologies for IMRT measurement-based verification QA: recommendations of AAPM Task Group No. 218. Med Phys. 2018 Apr;45(4):e53–e83. 10.1002/mp.12810 10.1002/mp.12810 29443390

[27] Solutions PTW. Radiation Medicine QA - Radiation Medicine Products from PTW, 2019 November.

[28] Synder JE, Hyer DE, Flynn RT, Boczkowski A, Wang D (2019). The commissioning and validation of Monaco treatment planning system on an Elekta Versa HD linear accelerator. J Appl Clin Med Phys.

[29] Georg D, Knöös T, McClean B. Current status and future perspective of flattening filter free photon beams. Med Phys. 2011 Mar;38(3):1280–93. 10.1118/1.3554643 10.1118/1.3554643 21520840

[30] From acceptance testing to linac go-live in nine days. ; 2020; https://focus.elekta.com/2020/10/from-acceptance-testing-to-linac-go-live-in-nine-days/ [accessed 27 November 2021].

[31] Cashmore J (2008). The characterization of unflattened photon beams from a 6 MV linear accelerator. Phys Med Biol.

[32] Almberg SS, Frengen J, Lindmo T (2012). Monte Carlo study of in-field and out-of-field dose distributions from a linear accelerator operating with and without a flattening-filter. Med Phys.

[33] Meshram MN, Pramanik S, Ranjith CP, Gopal SK, Dobhal R (2016). Dosimetric properties of equivalent-quality flattening filter-free (FFF) and flattened photon beams of versa HD linear accelerator. J App Clin Med Phys.

[34] Xu Z, Warrell G, Lee S, Colussi V, Zheng Y, Ellis R, Machtay M, Podder T. Assessment of beam-matched linacs quality/accuracy for interchanging SBRT or SRT patient using VMAT without replanning. *J Appl Clin Med Phys*. 2019 Jan;20(1):68–75. doi: 10.1002/acm2.12492. Epub 2018 Nov 7. PMID: 30402983. 10.1002/acm2.12492 PMC6333115 30402983

[35] Chang Z, Wu Q, Adamson J, Ren L, Bowsher J, Yan H, Thomas A, Yin FF (2012). Commissioning and dosimetric characteristics of TrueBeam system: Composite data of three TrueBeam machines. Med Phys.

[36] Hrbacek J, Lang S, Klöck S. Commissioning of photon beams of a flattening filter-free linear accelerator and the accuracy of beam modeling using an anisotropic analytical algorithm. Int J Radiat Oncol Biol Phys. 2011 Jul;15(4):1228–37. Epub 2010 Dec 2. 10.1016/j.ijrobp.2010.09.050 21129855

[37] Beyer GP. Commissioning measurements for photon beam data on three TrueBeam linear accelerators, and comparison with trilogy and clinac 2100 linear accelerators. J Appl Clin Med Phys. 2013 Jan;7(1):4077. 10.1120/jacmp.v14i1.4077 10.1120/jacmp.v14i1.4077 PMC5714054 23318395

[38] Tanaka Y, Akino Y, Mizuno H, Isono M, Masai N, Yamamoto T. Impact of detector selections on inter-institutional variability of flattening filter-free beam data for TrueBeam™ linear accelerators. J Appl Clin Med Phys. 2020 Jan;21(1):36–42. 10.1002/acm2.12766. Epub 2019 Nov 18. 10.1002/acm2.12766 PMC6964765 31738002

[39] Fogliata A, Fleckenstein J, Schneider F, Pachoud M, Ghandour S, Krauss H, Reggiori G, Stravato A, Lohr F, Scorsetti M, Cozzi L. Flattening filter free beams from TrueBeam and Versa HD units: evaluation of the parameters for quality assurance. Med Phys. 2016 Jan;43(1):205. 10.1118/1.4938060 10.1118/1.4938060 26745913

[40] Kragl G, afWetterstedt S, Knäusl B, Lind M, McCavana P, Knöös T, McClean B, Georg D. Dosimetric characteristics of 6 and 10MV unflattened photon beams. Radiother Oncol. 2009 Oct;93(1):141–6. 10.1016/j.radonc.2009.06.008. Epub 2009 Jul 9. 10.1016/j.radonc.2009.06.008 19592123

